# Analysis of hippocampal-dependent learning and memory behaviour in mice lacking *Nfix* from adult neural stem cells

**DOI:** 10.1186/s13104-018-3652-7

**Published:** 2018-08-06

**Authors:** Oressia Zalucki, Danyon Harkins, Lachlan Harris, Thomas H. J. Burne, Richard M. Gronostajski, Michael Piper

**Affiliations:** 1 The School of Biomedical Sciences, The University of Queensland, Brisbane, QLD 4072 Australia; 2 Queensland Brain Institute, The University of Queensland, Brisbane, 4072 Australia; 3 Queensland Centre for Mental Health Research, The Park Centre for Mental Health, Wacol, QLD 4076 Australia; 4 Department of Biochemistry, Program in Genetics, Genomics and Bioinformatics, Center of Excellence in Bioinformatics and Life Sciences, State University of New York at Buffalo, Buffalo, NY 14203 USA

**Keywords:** NFIX, Active place avoidance, Hippocampus, Learning and memory

## Abstract

**Objective:**

The active place avoidance task (APA) is a behavioural task used to assess learning and memory in rodents. This task relies on the hippocampus, a region of the cerebral cortex capable of generating new neurons from neural stem cells. In this study, to gain further insight into the behavioural phenotype of mice deficient in the transcription factor *Nfix*, a gene expressed by adult neural stem cells, we examined learning and memory parameters from the APA task that were not published in our original investigation. We analysed time to first and second shock, maximum path and time of shock avoidance, number of entries into the shock zone and time spent in the shock zone. We also assessed performance in the APA task based on sex.

**Results:**

We found mice deficient in *Nfix* displayed decreased latency to second shock compared to the control mice. *Nfix* deficient mice entered the shock zone more frequently and also spent more time in the shock zone. Our data provides further insights into the memory deficits evident in *Nfix* mutant mice, indicating these mice have a memory retrieval problem and may employ a different navigation strategy in the APA task.

**Electronic supplementary material:**

The online version of this article (10.1186/s13104-018-3652-7) contains supplementary material, which is available to authorized users.

## Introduction

The active place avoidance task (APA) is a well-established method for assessing learning and memory in rodents [1–5]. This task is designed to test spatial learning and memory in mice by evaluating the ability of the animal to avoid a fixed shock zone relative to external spatial cues, a task that requires the hippocampus. Mice are placed on a slowly rotating platform surrounded by external cues that are used to delineate the position of a fixed shock zone. As the platform is continually moving, mice must integrate conflicting positional information cues with the external positional cues to avoid the shock zone [2, 5].

Successful avoidance of the shock zone in the APA task is dependent on the generation of adult born neurons [6]. We have previously shown that in mice where the transcription factor nuclear factor one X (*Nfix*) is specifically ablated from adult hippocampal stem cells (referred to as *Nfix*^iNestin^ mice [7]), intermediate neuronal progenitors fail to differentiate, resulting in fewer adult-born hippocampal neurons generated, leading to deficits in learning and memory [7]. We showed that *Nfix*^iNestin^ mice receive significantly more shocks than control littermates in the APA task. Following our initial study, we further analysed our data, evaluating the latency to first and second shock. A decreased latency to first or second shock would be suggestive of a long-term memory retrieval problem [8], thus providing further insight into the learning and memory capabilities of *Nfix*^iNestin^ mice. In addition, we evaluated other learning and memory parameters from the APA task, including maximum time of shock avoidance, maximum path of shock avoidance, number of entries into the shock zone and time spent in the shock zone. We also analysed the performance of the mice based on sex, as our initial study utilised both male and female mice, which differs from previous studies using APA [2, 6, 8, 9]. Here, we show that there was no difference in the total number of shocks received by male or female mice in either control or *Nfix*^iNestin^ mice. We reveal *Nfix*^iNestin^ mice are capable of spatial learning, as demonstrated by their similar maximum time and distance travelled to avoid a shock compared to control mice. Rather, the disparate performance of *Nfix*^iNestin^ mice compared to controls was evidenced by more frequent entries into the shock zone, increased time spent in the shock zone and decreased latency to second shock. Thus, our analysis reveals *Nfix*^iNestin^ mice have altered learning capabilities, perhaps adopting different navigation strategies to perform the APA task.

## Main text

### Methods

#### Animals and tamoxifen treatment

Conditional *Nfix* mice (*Nfix*^f/f^) harbour *loxP* sites flanking exon 2 [10, 11]. These mice were crossed to the *nestin*-creER^T2^ strain [12] to generate homozygous *Nfix*^iNestin^ mice. All mice were maintained on a C57BL/6J genetic background, and had been backcrossed for 10 generations. Loss-of-function animals were homozygous *Nfix*^iNestin^ mice that were given tamoxifen dissolved in corn-oil. Control mice were *cre*-negative *Nfix*^Control^ mice treated with tamoxifen. Tamoxifen administration was performed as previously described [7]. Adult (8–10 week old) male and female mice were used in this study, assessed 45 days after tamoxifen administration. A total of 21 mice of each genotype was used. After behavioural testing, mice were anaesthetised, then were perfused transcardially with phosphate buffered saline (10 ml), then with 4% paraformaldehyde (30 ml).

#### Active place avoidance

The active place avoidance (APA) task was used to assess hippocampal-dependent spatial learning [4, 5]. Prior to testing, mice were handled by the researcher for 2–3 min for three consecutive days. The following day, mice were habituated to the behavioural arena for 5 min, with no shocks administered during this time (shock zone was inactive). The behavioural arena consisted of a grid floor (grid spacing 0.5 cm, total diameter 77 cm, Bio-Signal group). This grid is placed upon a base that has a motor and swivel to allow for the entire apparatus to rotate clockwise at 1 revolution/min. A 32 cm high clear plastic circular fence defined the perimeter of the arena. Four contrasting visual cues were placed equally on the walls of the testing room (approximate distance to visual cues from the arena perimeter is 45 cm). To commence a trial, the mouse was placed in the arena facing the wall directly opposite the designated shock zone, which did not change throughout the experiment. Once the mouse was placed in the arena the trial was started and rotation of the arena began. The position of the mouse in the arena was tracked by an overhead camera linked to Tracker software (Bio-Signal group, version 2.36). When the mouse entered the 60 degree shock zone, a 500 ms, 60 Hz, 0.5 mA shock was delivered through the grid floor via Shock Scrambler (Bio-Signal Group). Repeated shocks were administered every 1500 ms until the mouse left the shock zone. Each trial lasted 10 min, and was conducted for 5 consecutive days. Track Analysis software (Bio-Signal group, version 2.2) was used to analyse the recorded tracks for each mouse. All mice in our study received tamoxifen to control for effect of the drug on performance [13].

#### Statistical analyses

Two-way ANOVA was performed, with repeated-measures. Any significant main effect of genotype or day detected by two-way ANOVA was followed by multiple t-tests using a pooled estimate of variance where appropriate with *p* values reported in the text. Statistical analyses were performed in Prism 7.0 (Graphpad).

### Results

**Fig. 1:**
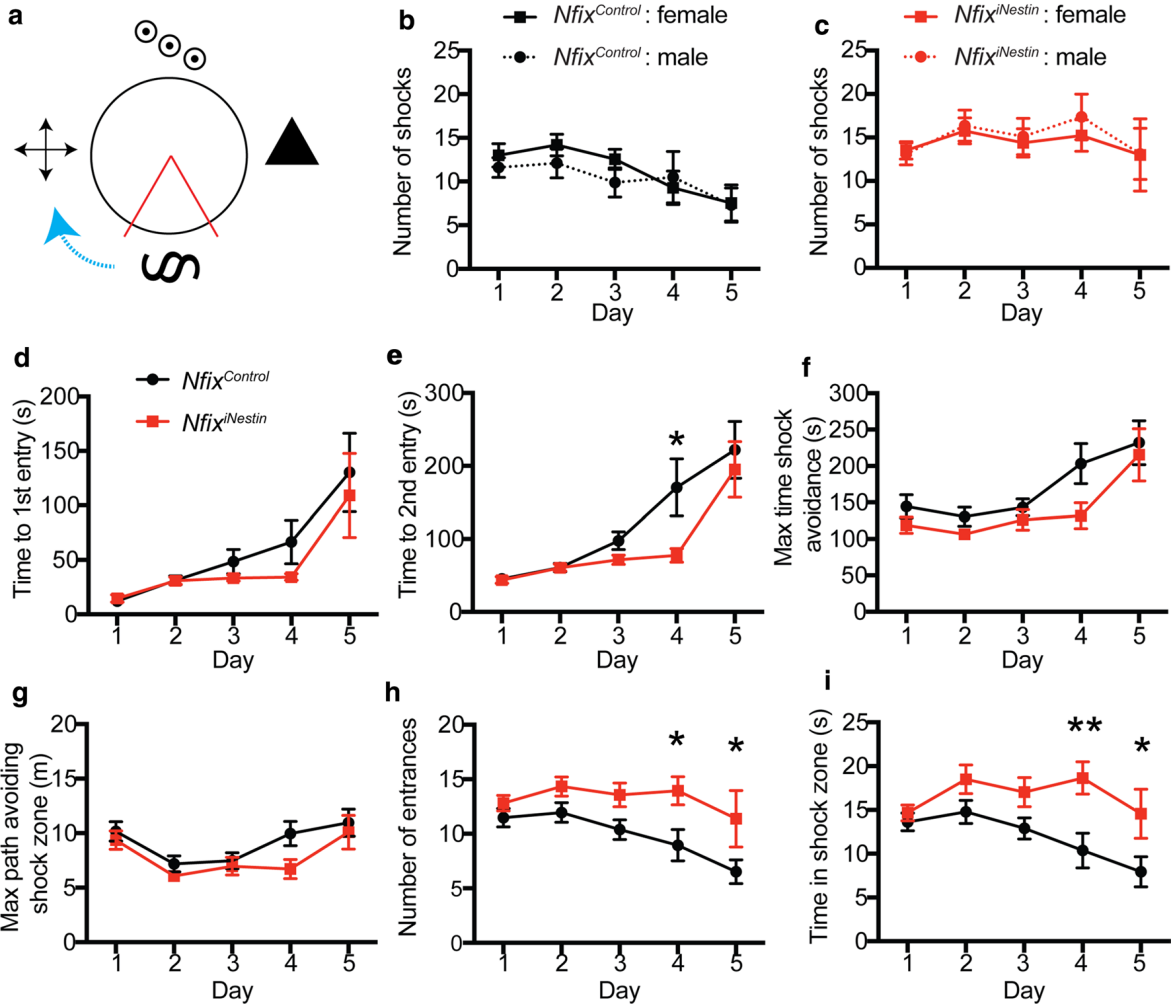
Behavioural analyses from the active place avoidance task. **a** Schematic of the active place avoidance (APA) testing arena. Four black and white visual cues are placed around the platform, which rotated clockwise (blue arrow). The shock zone location is shown in red. **b** The total number of shocks received by male (dashed line, n = 10) and female (solid line, n = 11) *Nfix*^Control^ mice in the APA task. **c** The total number of shocks received by male (red dashed line, n = 7) and female (red solid line, n = 14) *Nfix*^iNestin^ mice in the APA task. No significant differences were found in the number of shocks received by male and female mice of either genotype. **d** Time of first entry to the shock zone was not different in *Nfix*^Control^ mice (black) and *Nfix*^iNestin^ mice (red) during the APA task. **e** Time to second entrance into the shock zone was significantly delayed in *Nfix*^Control^ mice (black) compared to *Nfix*^iNestin^ mice (red) on day 4 of the APA task. Maximum time shock avoidance (**f**) and maximum path of shock avoidance (**g**) during the APA task was not significantly different between *Nfix*^Control^ mice (black) and *Nfix*^iNestin^ mice (red). **h** The number of entries into the shock zone was significantly higher in *Nfix*^iNestin^ (red) mice on days 4 and 5 of the APA task compared to *Nfix*^Control^ mice (black). **i** Total time spent in the shock zone was significantly longer for *Nfix*^iNestin^ (red) mice on days 4 and 5 of the APA task compared to *Nfix*^Control^ mice (black). **p < 0.01, *p < 0.05. All graphs depict mean ± s.e.m; n = 21 mice per genotype in (**d**–**i**)

In rodents, the generation of adult born hippocampal neurons is required for learning and memory. We previously reported that *Nfix*^iNestin^ mice generate fewer mature dentate granule neurons and consequently display learning deficits in a hippocampal-dependent learning and memory task [7]; (Fig. 1a). We found across the 5 days of the APA task, *Nfix*^iNestin^ mice consistently received the same number of shocks, in contrast to control mice, who received significantly fewer shocks on days 4 and 5. Our initial analysis pooled mice from both sexes. Since many previous studies utilising the APA task typically use only one sex of mouse [2, 6, 8, 9], we therefore first explored if there were any differences in performance in the APA task based on the sex of the mice. When analysing the total number of shocks, we found no sex differences across the 5 days of testing for either control (Fig. 1b) or *Nfix*^iNestin^ mice (Fig. 1c).

Our initial description of the learning and memory phenotype of *Nfix*^iNestin^ mice only reported the total number of shocks received across the 5 days of the APA task. Whilst this analysis provided a measure of the cumulative performance of the mice in the task, it did not specifically assess the long-term encoding and retrieval of spatial memory, which is better addressed by examining latency to first and second shock [8]. We therefore further probed the learning and memory deficit apparent in the *Nfix*^iNestin^ mice by analysing the latency to these shocks. For latency to first shock, we found no difference between the genotypes (Fig. 1d). For latency to second shock, we found improved performance in control mice (*F*_*1, 40*_ = 9.34, *p *= .003) on day 4 of the APA task compared to *Nfix*^iNestin^ mice (*p *= .016, Fig. 1e). Interestingly, we found female *Nfix*^iNestin^ mice perform better than their male counterparts for both time to first (*p *= .008, Additional file 1: Figure S1B) and second shock on day 5 (*p *< 0.001, Additional file 1: Figure S1D).

To further evaluate the learning and memory capabilities of *Nfix*^iNestin^ mice, we analysed other parameters assessed in the APA task, including the maximum time shock avoidance, the maximum path implemented to avoid a shock, number of entries into the shock zone and time spent in the shock zone. We found no significant differences between control and *Nfix*^iNestin^ mice in the maximum time of shock avoidance (Fig. 1f) and maximum path of shock avoidance (Fig. 1g), suggesting both genotypes form a spatial memory of the location of the shock zone. Why then do *Nfix*^iNestin^ mice consistently receive the same number of shocks across the days of the APA task? We found the total number of entries into the shock zone was significantly higher in *Nfix*^iNestin^ mice (*F*_*1*_, _*40*_ = 9.15, *p * = .0043) on days 4 (*p * = .031) and 5 (*p * = .031) of the task compared to controls (Fig. 1h), and that *Nfix*^iNestin^ mice spend significantly more time in the shock zone (*F*_*1, 40*_ = 10.61, *p * = .023) on days 4 (*p * = .0033) and 5 (*p * = .024) compared to control mice (Fig. 1i). We found no differences between male and female mice of either genotype for these remaining APA parameters (Additional file 1: Figure S1E–L).

### Discussion

The APA task is designed to probe spatial learning and memory. Successful avoidance of the shock zone in the APA task relies in part on adult hippocampal neurogenesis [5, 6, 9, 14]. Adult born hippocampal neurons are generated from neural stem cells which reside in the dentate gyrus of the hippocampus [15]. We have previously shown that *Nfix* is expressed by adult neural stem cells, and that in *Nfix*^iNestin^ mice, where *Nfix* is specifically ablated from adult neural stem cells, fewer adult born neurons are produced and these mice consequently show learning and memory deficits [7]. However, lacking from this study was an appreciation of the extent of the learning and memory deficit, including if there was a memory encoding and retrieval problem. Here, we extended our behavioural analysis of *Nfix*^iNestin^ mice, analysing latency to first and second shock, maximum time shock avoidance, maximum path shock avoidance, number of entries into the shock zone and time spent in the shock zone. Our initial experimental design utilised both male and female mice, whereas many previous APA investigations only use one sex [2, 6, 8, 9]. We could therefore partition the data based on sex, and ask if there were any sex differences apparent in performance in the APA task. We show that there was no difference in the total number of shocks received by male and female mice in either control or *Nfix*^iNestin^ mice. Whilst using only one sex of animal in behavioural studies reduces potential sources of variability, this may not always be feasible when working with transgenic mice. Indeed, a recent commentary highlighted the over-reliance on male mice used for experiments, and how this might potentially bias and affect experimental outcomes and impact the translatability of research to human populations [16]. The results of our study suggest that pooling male and female mice for the APA task could be considered a feasible option, as others have done when working with transgenic mice [17].

We previously reported that *Nfix*^iNestin^ mice move at a similar speed and distance to controls in the APA task [7]. Here we show that the maximum time and path of shock avoidance was similar between the two genotypes, indicating that the reduced performance of *Nfix*^iNestin^ mice in the APA task is not related to movement differences. Furthermore, it is unlikely that altered pain perception can explain our results, as our genetic manipulation of knocking out *Nfix* from adult neural stem cells is performed in adult mice and is highly specific, and *Nfix*^iNestin^ mice are responsive to tail pinch [7]. Here, we show that the learning and memory deficit in *Nfix*^iNestin^ mice likely relates to their inability to escape the shock zone, with *Nfix*^iNestin^ mice entering the shock zone more frequently and spending more time in the shock zone than control mice. This may be a reflection of the different spatial navigation strategies used by *Nfix*^iNestin^ mice in the APA task. The APA task relies on allocentric spatial navigation, where the rotating arena requires the mouse to continually integrate visual cues to orient itself relative to the room to avoid the fixed shock zone location [2]. In contrast, egocentric navigation relies on cues generated by self-movement, such as proprioceptive cues or cues from optic or auditory flow. Relying on egocentric cues to predict the shock zone could result in poor performance in the APA task, and may explain why *Nfix*^iNestin^ mice spend more time in the shock zone and receive more shocks compared to controls. Whilst previous work suggests male mice perform better in allocentric navigation tasks and females in egocentric navigation tasks [18, 19], we found no differences in maximum time or path of shock avoidance and time spent in the shock zone for control and *Nfix*^iNestin^ mice based on sex. Female *Nfix*^iNestin^ mice did show improved long term memory capabilities compared to their male counterparts, with an increased latency to first and second shock, but this did not translate into fewer shocks received. To provide a more comprehensive understanding of spatial navigation in *Nfix*^iNestin^ mice, future investigations should analyse other forms of spatial learning, including egocentric learning and memory tasks, such as the Cincinnati water maze [20].

The results from our study and previous work [7] affirm the importance of *Nfix* in broad aspects of adult neural stem cell biology, from their production and survival to its functional importance in learning and memory. Understanding these diverse roles of *Nfix* will allow insight into human congenital disorders caused by mutations in *NFIX*, such as Marshall-Smith syndrome and Malan syndrome [21, 22]. These patients have a degree of intellectual disability, including short-term and working memory deficits [23]. Whilst no gross hippocampal abnormalities are reported in these patients [24], subtle changes in hippocampal morphology and altered network dynamics resulting from fewer adult born neurons could contribute to the neurological deficits seen in these patients.

## Limitations

Our analysis of learning and memory using the APA task assesses hippocampal-dependent behaviour. Other behavioural assays would provide a more thorough investigation of other forms of spatial learning and memory.

## Additional file

**Additional file 1: Figure S1.** Comparison of male and female mice for additional behavioural analyses from the active place avoidance task. (**A**) Time of first entry into the shock zone was not different in male (dashed line) and female (solid line) *Nfix*^Control^ mice. (**B**) Time to first entrance into the shock zone was significantly delayed in female *Nfix*^iNestin^ mice (solid line) compared to male *Nfix*^iNestin^ mice (dashed line) on day 5 of the APA task. (**C**) Time of second entry into the shock zone was not different in male (dashed line) and female (solid line) *Nfix*^Control^ mice. (**D**) Time to second entrance into the shock zone was significantly delayed in female *Nfix*^iNestin^ mice (solid line) compared to male *Nfix*^iNestin^ mice (dashed line) on day 5 of the APA task. Maximum time shock avoidance was not significantly different between male (dashed line) and female mice (solid line) for *Nfix*^Control^ mice (black, **E**) or *Nfix*^iNestin^ mice (red, **F**). Maximum path shock avoidance was not significantly different between male (dashed line) and female mice (solid line) for *Nfix*^Control^ mice (black, **G**) or *Nfix*^iNestin^ mice (red, **H**). The number of entries into the shock zone was not significantly different between male (dashed line) and female mice (solid line) for *Nfix*^Control^ mice (black, **I**) or *Nfix*^iNestin^ mice (red, **J**). Time spent in the shock zone was not significantly different between male (dashed line) and female mice (solid line) for *Nfix*^Control^ mice (black, **K**) or *Nfix*^iNestin^ mice (red, **L**). ** *p* < 0.01, *** *p* < 0.001. All graphs depict mean ± s.e.m; n = 10 male *Nfix*^Control^ mice, n = 11 female *Nfix*^Control^ mice, n = 7 male *Nfix*^iNestin^ mice and n = 14 female *Nfix*^iNestin^ mice.

## Abbreviations

APA: active place avoidance
NFIX: nuclear factor one X
