# Supplementary figures and images for: Analysis of hippocampal-dependent learning and memory behaviour in mice lacking Nfix from adult neural stem cells
Source: BMC Res Notes. 2018 Aug 6;11:564. doi: 10.1186/s13104-018-3652-7 (PMC6080370; doi:10.1186/s13104-018-3652-7)

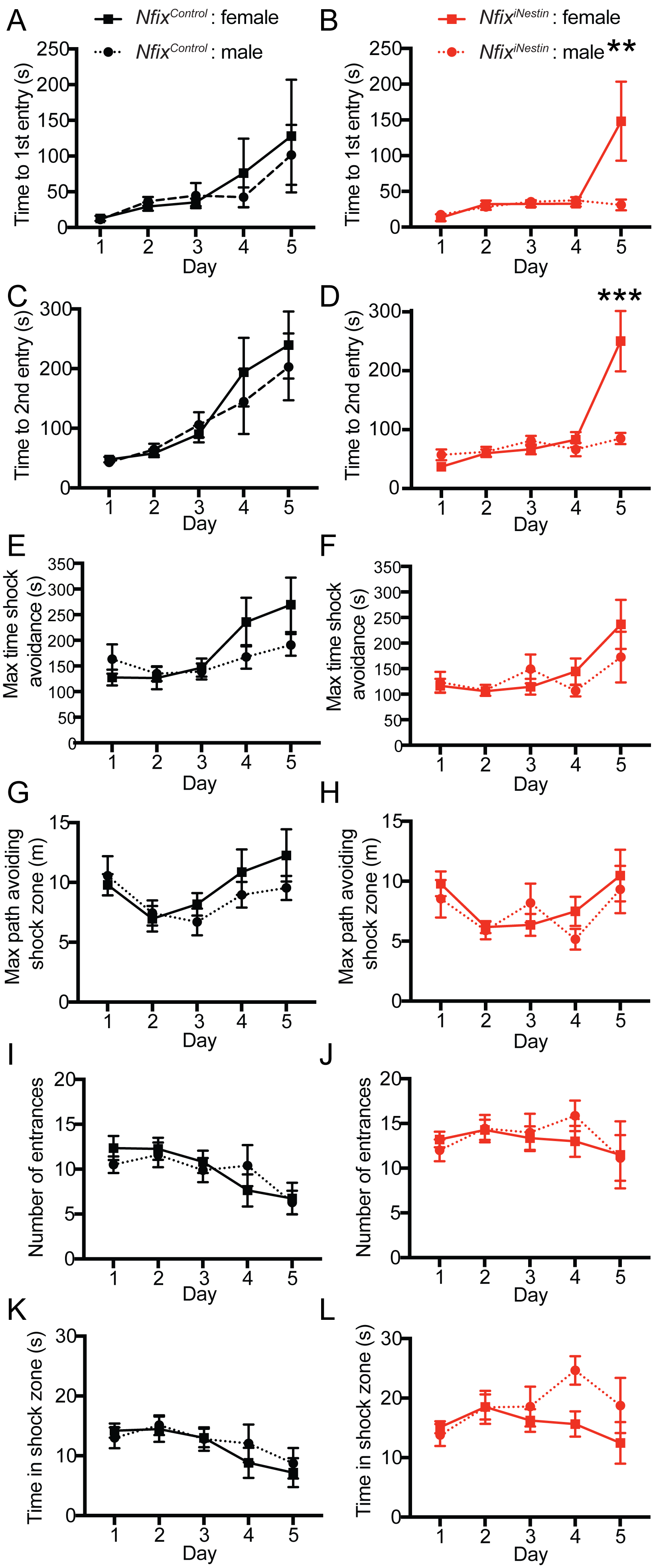

Supplement: Supplementary file 1 — Additional file 1: Figure S1. Comparison of male and female mice for additional behavioural analyses from the active place avoidance task. (A) Time of first entry into the shock zone was not different in male (dashed line) and female (solid line) NfixControl mice. (B) Time to first entrance into the shock zone was significantly delayed in female NfixiNestin mice (solid line) compared to male NfixiNestin mice (dashed line) on day 5 of the APA task. (C) Time of second entry into the shock zone was not different in male (dashed line) and female (solid line) NfixControl mice. (D) Time to second entrance into the shock zone was significantly delayed in female NfixiNestin mice (solid line) compared to male NfixiNestin mice (dashed line) on day 5 of the APA task. Maximum time shock avoidance was not significantly different between male (dashed line) and female mice (solid line) for NfixControl mice (black, E) or NfixiNestin mice (red, F). Maximum path shock avoidance was not significantly different between male (dashed line) and female mice (solid line) for NfixControl mice (black, G) or NfixiNestin mice (red, H). The number of entries into the shock zone was not significantly different between male (dashed line) and female mice (solid line) for NfixControl mice (black, I) or NfixiNestin mice (red, J). Time spent in the shock zone was not significantly different between male (dashed line) and female mice (solid line) for NfixControl mice (black, K) or NfixiNestin mice (red, L). ** p < 0.01, *** p < 0.001. All graphs depict mean ± s.e.m; n = 10 male NfixControl mice, n = 11 female NfixControl mice, n = 7 male NfixiNestin mice and n = 14 female NfixiNestin mice. [file 13104_2018_3652_MOESM1_ESM.tif]
